# The Neural Substrate and Functional Integration of Uncertainty in Decision Making: An Information Theory Approach

**DOI:** 10.1371/journal.pone.0017408

**Published:** 2011-03-09

**Authors:** Joaquín Goñi, Maite Aznárez-Sanado, Gonzalo Arrondo, María Fernández-Seara, Francis R. Loayza, Franz H. Heukamp, María A. Pastor

**Affiliations:** 1 Neuroimaging Laboratory, Department of Neurosciences, Center for Applied Medical Research, University of Navarra, Pamplona, Spain; 2 IESE Business School, University of Navarra, Barcelona, Spain; University of Maribor, Slovenia

## Abstract

Decision making can be regarded as the outcome of cognitive processes leading to the selection of a course of action among several alternatives. Borrowing a central measurement from information theory, Shannon entropy, we quantified the uncertainties produced by decisions of participants within an economic decision task under different configurations of reward probability and time. These descriptors were used to obtain blood oxygen level-dependent (BOLD) signal correlates of uncertainty and two clusters codifying the Shannon entropy of task configurations were identified: a large cluster including parts of the right middle cingulate cortex (MCC) and left and right pre-supplementary motor areas (pre-SMA) and a small cluster at the left anterior thalamus. Subsequent functional connectivity analyses using the psycho-physiological interactions model identified areas involved in the functional integration of uncertainty. Results indicate that clusters mostly located at frontal and temporal cortices experienced an increased connectivity with the right MCC and left and right pre-SMA as the uncertainty was higher. Furthermore, pre-SMA was also functionally connected to a rich set of areas, most of them associative areas located at occipital and parietal lobes. This study provides a map of the human brain segregation and integration (i.e., neural substrate and functional connectivity respectively) of the uncertainty associated to an economic decision making paradigm.

## Introduction

Consider an economic decision paradigm with two options. The first option (A) is constant and consists of winning 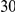 euros after 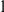 month with 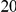
% of probability, while the second option (B) can consist, for instance, of winning the same amount of money after 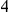 months with 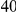
% of probability. Some people would prefer the first -closer in time but riskier- option and some others would prefer the second -delayed in time but safer- option. When varying the probability and the time of option B, one could find a task configuration where both options are evaluated as highly similar in terms of attractiveness. This kind of situation gives rise to a decision conflict. Different task configurations might produce heterogeneous decision patterns covering from a predominance of option A to a predominance of option B. Briefly, task configurations that produce a predominant answer (either option A or B) can be characterized by a low uncertainty, while task configurations with a balanced number of A and B outcomes can be characterized by a high uncertainty. The variability of the outcomes comes from within- and inter-subject variabilities. The former happens when decisions of a subject for certain configuration are not self-consistent and the latter happens when different subjects provide opposed decisions.

How can the level of conflict in a decision be evaluated? This question has received increasing attention in the last decade. Prediction paradigms, where participants have to anticipate an outcome have been the norm. In such paradigms, the level of ambiguity of the experiment is controlled, manipulating either the information the subject used to correctly make the prediction [1], [2] or the probability of success [2]–[6]. Consequently in these studies the ambiguity level was proposed a priori during the design stage. However, it has been shown that sometimes participants behavior does not necessarily correspond to that inferred from the probability of success [7]. In two of the earliest studies, participants had to advance the color or the suit of a card [3] or whether the next card was bigger or lower than the previous one [4]. This permitted the comparison between low and high difficulty guessing. Prefrontal areas, but also the anterior cingulate, were more related to trials with high difficulty. In other studies [7], [8] participants predicted the appearance of stimuli. Prefrontal, parietal and thalamic areas were active during such task. Volz et al. [1], [2], [5] presented pairs of alien comic figures and subjects had to infer which figure would win in a fictional fight. In one of the experiments there was an unknown probability of winning for each pair of figures that had to be learned as the experiment advanced. In the other experiment there were a set of rules that marked which figure won each time. The level of uncertainty of the experiment was manipulated by varying the degree of knowledge of the winning rules provided to the participants. Although there were minor differences in brain activation between the two paradigms, a fronto-median cluster correlated with the degree of uncertainty independently of the paradigm used. Huettel et al. also found a frontomedian activation when processing uncertainty in a paradigm where visual cues helped to predict the following answer [6]. In a more recent article [9], male subjects were required to discriminate attractiveness between pairs of women faces. Each picture had been rated previously by another group of participants, allowing to estimate and control the level of decision conflict. While all these studies associate pre-frontal and or fronto-median areas to the processing of conflict, a role in uncertainty management has also been assigned to the cerebellum [10].

As shown above, the concepts of certainty/uncertainty have been commonly used in decision making studies and most of their quantifications have been represented by either theoretical probability distributions or by empirical relative frequencies. Interestingly, an uncertainty descriptor that can be quantified from any probability distribution is the central measurement of information theory. In information theory, Shannon entropy [11] (denoted by 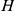) measures the amount of information or uncertainty contained in a message (usually measured in bits). Its use in decision making tasks has been scarce [12], [13] and mostly focused on the uncertainty of task-related probabilistic events [14]–[16] and not on the decisions of the subjects. However, the close relationship between Shannon's concept of information and the psychological concept of uncertainty has been pointed out [6]. Briefly, 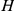 for random variables with 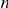 possible values has two main properties. First, 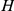 is 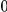 bits if and only if all the values contained in the message are the same (i.e, the outcome is completely certain). Second, 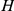 is maximum when the frequency of values in the message is equal, resulting in 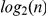 bits. Intuitively, a sequence of flipping a perfect coin would have maximum entropy (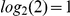 bit) while a two-tails coin would have the minimum entropy (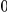 bits). Going back to our economic decision task, let us consider that we aim to transmit within a message (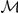) the decisions of all participants for a certain task configuration (i.e. specifying the probability and time of option B). Such message will be formed by a finite sequence of symbols with values A or B indicating the options selected. What would be the uncertainty of the message? On the one hand, a message with the decisions for a very easy task configuration would be constant (either 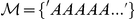 or 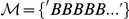) and thus the uncertainty associated to it would be 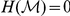 bits. On the other hand a message formed by the decisions for a very difficult task configuration would be, for instance, 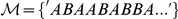 and thus the uncertainty associated to it would be 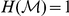 bit. Messages obtained from other task configurations would produce intermediate values of uncertainty within the range 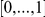.

The aim of this study is to introduce the concept of Shannon entropy in decision making paradigms as a decision uncertainty descriptor of the task and to map the functional fingerprint of such uncertainty using an economic decision task under different configurations of probability and time. To achieve this, decision outcomes and fMRI BOLD data were analyzed in three steps. Firstly, Shannon entropy concept was used to characterize the decision uncertainty associated to each task configuration in terms of within- inter- and pooled-variabilities. Multi-linear regression analyses revealed that pooled-entropy was the best predictor of the response times and was used to characterize the uncertainty associated to each task configuration. Secondly, these pooled-entropy values were used as a neural correlate with BOLD activity in order to obtain brain areas codifying uncertainty. Thirdly, the psycho-physiological interactions (PPI) paradigm and a conjunction analysis were employed to study the functional integration of the uncertainty codification, i.e., which brain areas gained functional connectivity as the entropy associated to the task configurations increased.

## Results

### Behavioral results

During the scanning sessions performed, participants answered 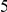 times to each of the 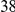 different task configurations presented in a pseudo-random order. Each task was constituted by two options and each answer consisted of making a binary choice between them. For 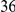 out of the 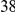 task configurations, there was a constant option (A) which consisted of winning 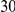 euros after 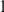 month with 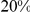 of probability. In those cases the alternative option (B) was different at each task configuration by varying the time from 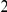 to 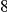 months and the reward probability from 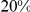 to 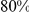. Two additional configurations were included in which both options A and B varied (see methods for a detailed explanation).

In our decision making experiment, uncertainty associated to a task configuration is intrinsically related to the variability of the decisions reported for such task. The decision sets of each task configuration (i.e. the collection containing all the decisions reported by the subjects for a specific task configuration) contain two different sources of variability that might contribute to quantify the level of uncertainty. On the one hand, a high within-subject variability reveals lack of self-consistency during the 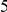 responses made by a participant for certain task configuration. On the other hand, a high inter-subject variability reveals the existence of opposed preferences among individuals. Both factors add evidence of a task being difficult, i.e. a task with associated high uncertainty. Furthermore we hypothesized that the entropy of the *pooled* variability containing both inter-subject and within-subject variabilities might be an appropriate descriptor of such task uncertainty level. Hence we quantified the entropy of each task in terms of within-subject variability (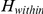), inter-subject variability (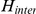) and pooled variability (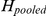).

**Figure 1:**
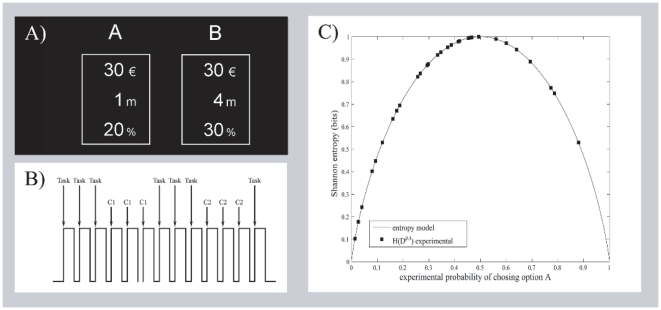
Overview of the decision making paradigm. **A. Visual presentation.** Example of visual presentation with two options shown to the participants during the decision-making task. This presentation corresponds to the task configuration 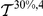. **B. Presentation design.** The decision-making trials were presented in blocks of three and were interleaved alternatively with one of the different controls (C1, C2), which also appeared in blocks of three. **C. Shannon entropy.** Continuous line stands for the entropy model with respect to the probability (dichotomous variable) and squares refer to experimental entropy values of the pooled decisions, 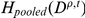, made at different task configurations, 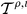. Note that entropy model is symmetric to the probability. It reaches low values when the variable under study takes most of the times either one value or the other, and reaches its maximum when the random variable takes each of the 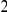 possible values with 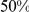 of probability.

**Figure 2:**
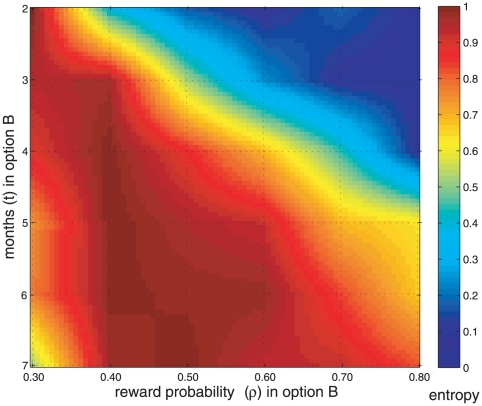
Shannon entropy map (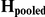) associated to the decision task 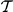. Reward probability (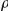) and time (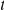) axis characterize option B {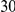 euros,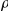, 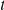} and define each task configuration 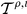, since option A is constant {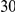 euros, 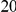
% 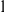 month}. Vertices formed by dotted lines correspond to actual evaluated task configurations and intermediate points are the result of a bi-dimensional interpolation process. Two additional task configurations with no constant option are not included in this figure.

Individual entropy maps of 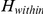 can be seen at Figure S1, where the presence of highly consistent subjects (such as 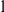, 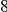, and 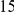) and lowly consistent subjects (such as 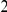, 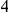 and 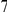) can be observed. Tables S1 and S2 summarize the entropy values corresponding to 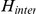 and 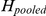 respectively. These two descriptors show a qualitative similar behavior. Values corresponding to 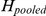 displayed in Figure 1C may be presented as an interpolated entropy map (Figure 2) which summarizes the effects of probability and time dimensions on the uncertainty of the decisions. Results in a numerical format are shown in Table S2. Axis 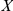 and 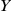 respectively determine the time (months) and the reward probability of every option 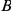. Therefore each point unequivocally represents one task configuration. An entry at row 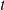 and column 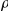 of Table S2 describes the pooled uncertainty of the task configuration whose options presented were 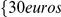,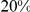,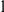 month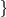 and 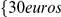,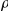,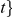, i.e., the constant and alternative options respectively. Covering the range of a value delimited variable (Shannon entropy 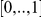 in our case) is specially relevant in order to have accurate results when using it as a neural correlate. The set of task configurations selected for our experiment produced a heterogeneous set of uncertainty values of 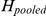 within the range 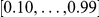. For instance, we identified low-uncertainty configurations such as 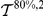 and 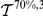, intermediate uncertainty configurations such as 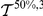 and 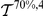 and high uncertainty configurations such as 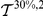 and 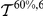.

One of the most common indicators of difficulty is the response time (RT). The average RT per subject per task 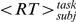 was used as the dependent variable in three multi-linear regression analyses including as independent variables the average response time of each subject 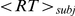 and one of the entropies at a time (within- inter and pooled-entropies). Results shown in Table 1 revealed that, being the three entropies significant factors, 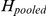 was the best predictor of response times and hence was used to characterize the entropy associated to each task. Additional evidence of the appropriateness of this approach was obtained an analogous the multi-linear regression analysis performed only on consistent decisions (see Table 1). We selected, for each subject and for each task configuration, only those sets of 5 responses that had been fully consistent (i.e. always A or always B). This corresponds to zero entropy values at the individual entropy maps of Figure S1. This subset of decisions was used in the last model in Table 1 to show that, even in this consistent data subset, 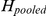 significantly contributed to predict 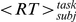.

**Table 1:** Multi-linear regression analyses.

Model	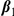	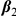	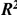
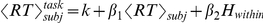	0.613	0.319	0.485
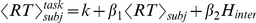	0.619	0.322	0.487
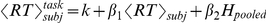	0.619	0.357	0.510
Model (consistent decisions only, i.e. where 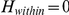 )			
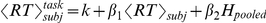	0.639	0.322	0.505

Evaluation of the three entropy measurements (within-subject, inter-subject and pooled) with respect to average response times per subject per task 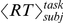. Models include the average response time of each subject 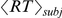 during the experiment and a constant 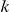. Beta values correspond to the standardized coefficients. 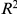 reflects the fraction of variance of 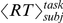 explained by the model. Results indicate that, when 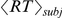 is fixed, 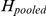 is the best descriptor in order to explain 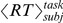. This model containing the term 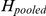 had also the highest . Even when analyzing only the consistent decisions per subject per task, 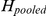 had a significant predictive capacity and the model was able to explain about half of the variability of 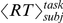.

### Neural correlate between BOLD signal and uncertainty

**Figure 3:**
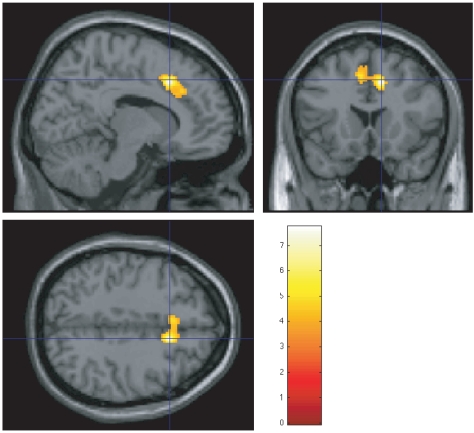
Cluster codifying uncertainty. This is cluster 1 at Table 2. It includes parts of the right MCC and of the pre-SMA(bilateral) obtained by using Shannon entropy (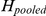) of decision sets as a neural correlate (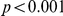 uncorrected, 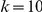). MNI Coordinates indicated by intersecting blue lines are [12 18 42] and correspond to global maxima correlation, which is located at the right MCC. A second cluster located at left thalamus was also found. Figure S3 shows coronal and sagital views of the two clusters.

We evaluated the set of 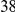 uncertainties 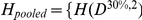, …, 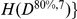, one from each task configuration, as a neural correlate. This allowed us to test which brain areas showed during every task a BOLD activity that codifies the decision uncertainty quantified for each task. The regression analysis yielded two clusters (see Table 2). The largest one (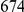 voxels, Figure 3) includes parts of the right middle cingulate cortex (MCC), of pre-supplementary motor area (pre-SMA, bilateral) and a small part of the left superior medial gyrus. A second cluster (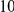 voxels) was found in the left thalamus. It includes part of mediodorsal (MD) nucleus and ventral anterior (VA) that project to the pre-frontal cortex [17]. These two clusters showed also greater activation during the decision making tasks (DM) than during the motor action control (C2). The DM
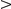
C2 t-contrast map (Figure S2) shows those areas with a higher activation during DM with respect to C2. Figure S3 shows coronal and sagital views of the two clusters.

**Table 2:** Neural substrate of uncertainty.

Lobe	Anatomical area	Side	MNI coordinates			t-value	Cluster (size)
			x	y	z		
Frontal	Middle Cingulate Cortex	R	12	18	42	7.72	1 (674)
	pre-SMA	L,R	−8	8	52	6.07	1 (674)
	Superior Medial Gyrus	L	−6	24	38	5.45	1 (674)
Thalamus	MD nucleus and VA	L	−10	−8	−2	4.04	2 (10)

Height threshold: t-value = 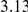, 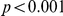 uncorrected.

Extent threshold: 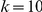 voxels.

Clusters codifying uncertainty of the economic decision task, i.e., with BOLD signal positively correlated with the entropy of the decisions produced at each task configuration.

### Functional integration of uncertainty

**Figure 4:**
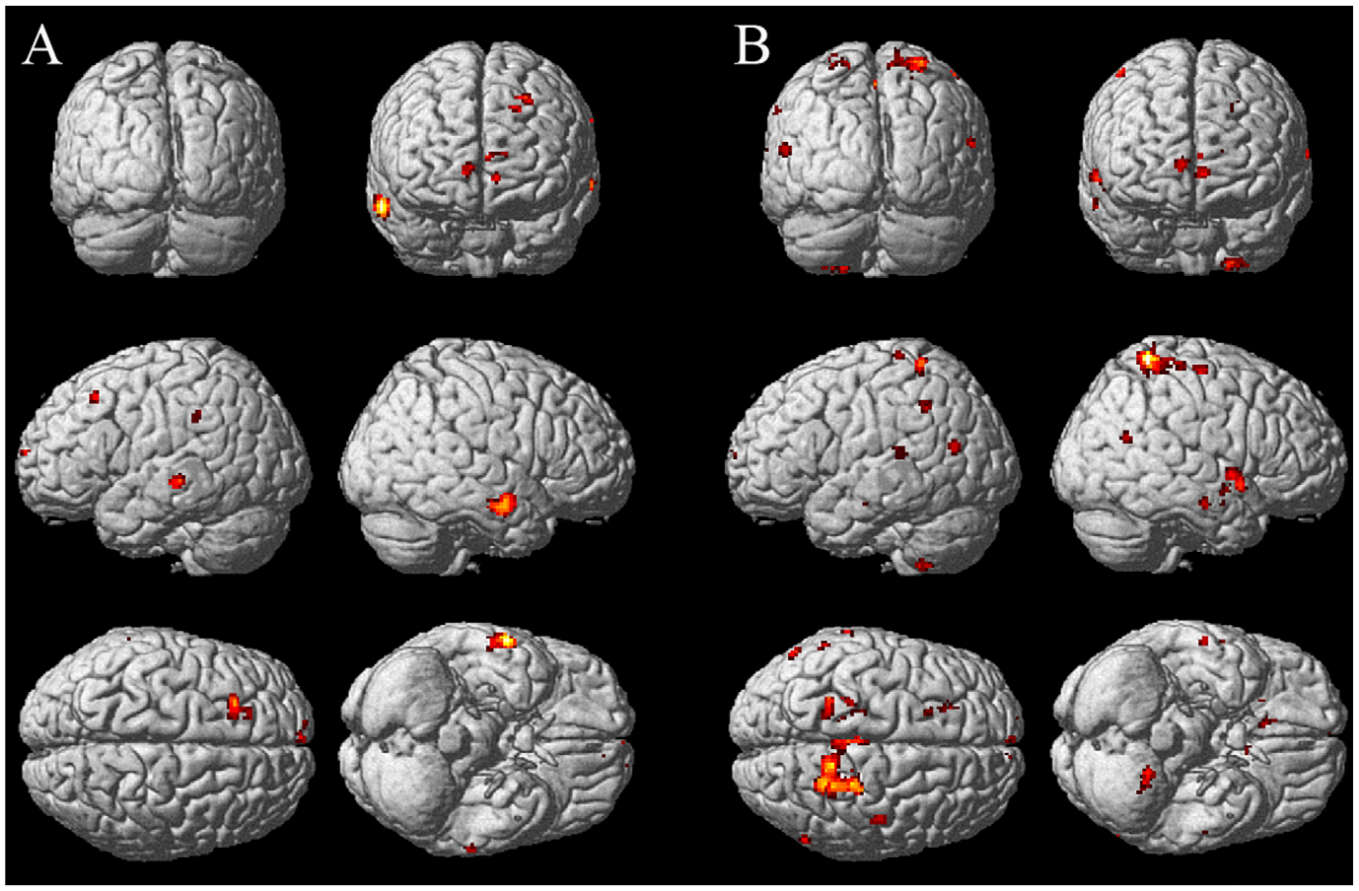
Functional integration of uncertainty. A. Clusters that increment functional connectivity with right MCC as entropy 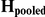 increases (PPI analysis). B. Clusters that increment functional connectivity with pre-SMA(bilateral) as entropy 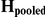 increases. This is the result of a conjunction analysis of the PPIs seeded in left and right pre-SMAs. The psychological variable was the Shannon entropy (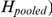 of the decisions associated to each task configuration.

Enhanced connectivity was detected by means of PPI analyses seeded in those areas of the largest cluster that codified uncertainty. This cluster includes MCC(right) and pre-SMA(left and right). A conjunction analysis was applied to the pre-SMA PPIs in order to obtain common increased connectivities to both pre-SMAs. These analyses (see methods for a detailed explanation) allowed us to identify areas that gained connectivity with MCC(right) or with pre-SMA(bilateral) as the decision uncertainty increased in the economic decision making task. The PPI seeded in the MCC(right) identified clusters located in *frontal lobe* -left middle frontal gyrus, left superior frontal gyrus, superior medial gyrus (bilateral) and left middle orbital gyrus-, and *temporal lobe* -middle temporal gyrus (bilateral)-. A summary of areas is listed in Table 3 and shown in Figure 4A. Clusters are listed in Table S3.

**Table 3:** Functional integration of uncertainty focused on right MCC.

Lobe	Anatomical area	Side	MNI coordinates			t-value
			x	y	z	
Frontal	Middle Frontal Gyrus	L	−24	24	46	5.35
	Superior Medial Gyrus	L,R	10	62	2	5.20
	Middle Orbital Gyrus	L	−8	54	−2	5.06
	Superior Frontal Gyrus	L	−16	30	40	4.68
Temporal	Middle Temporal Gyrus	L,R	62	−6	−24	5.91

Height threshold: t-value = 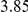, 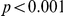 uncorrected.

Extent threshold: 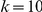 voxels.

MNI coordinates of seed at right MCC: [12 18 42].

Areas showing functional connectivity with right MCC as the entropy increases (PPI analysis). The psychological variable was the Shannon entropy of the decisions associated to each task configuration.

The conjunction analysis of PPIs seeded in left and right preSMAs identified areas located in *frontal lobe* -MCC (bilateral), paracentral lobule (bilateral), left middle frontal gyrus, left anterior cingulate cortex (ACC), right superior medial gyrus, right precental gyrus and left superior frontal gyrus -, *temporal lobe* - right temporal pole, middle temporal gyrus (bilateral) and right superior temporal gyrus -, *parietal lobe* - right precuneus, right superior parietal lobule and right postcentral gyrus -, *subcortical structures* - left caudate nucleus and left anterior thalamus -, *cerebelum*- left VIII -, and *insular* - right posterior insula -. A summary of areas is listed in Table 4 and shown in Figure 4B. Clusters are listed in Table S4.

**Table 4:** Functional integration of uncertainty focused on bilateral pre-SMA.

Lobe	Anatomical area	Side	MNI coordinates			t-value
			x	y	z	
Frontal	Superior Medial Gyrus	L,R	10	60	4	4.12
	Precentral Gyrus	R	46	−14	62	4.11
	Middle Cingulate Cortex	L,R	−2	−28	46	3.81
	Anterior Cingulate Cortex	L	−4	50	0	3.83
	Paracentral Lobule	R	14	−32	50	3.64
	Middle Frontal Gyrus	L	−20	22	44	3.62
	Superior Frontal Gyrus	L	−18	14	48	3.62
Temporal	Temporal Pole	R	62	4	−2	4.18
	Middle Temporal Gyrus	R	60	−12	−20	3.94
	Superior Temporal Gyrus	R	58	−60	22	3.80
Parietal	Precuneus	R	12	−46	62	4.51
	Superior Parietal Lobule	L	−26	−46	66	3.84
	Postcentral Gyrus	L	−26	−32	72	3.82
Insular	Posterior Insula	R	40	−6	−8	4.34
Cerebellum	VIII	L	−20	−46	−56	5.03
Basal ganglia	Caudate Nucleus	L	−6	14	−4	4.31
Thalamus	Pulvinar	L	−14	−34	2	3.80

Height threshold: t-value = 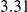,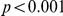 uncorrected.

Extent threshold: 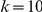 voxels.

MNI coordinates of seed at left pre-SMA: [−8 8 52].

MNI coordinates of seed at right pre-SMA: [6 12 54].

Areas showing functional connectivity with pre-SMA as the entropy increases. This is the result of a conjunction analysis of the PPIs with seeded in left and right pre-SMAs. The psychological variable was the Shannon entropy of the decisions associated to each task configuration.

These results indicate that, as the entropy associated to the decision making task increases, clusters mostly belonging to the associative cortex and located at frontal, temporal and parietal lobes get involved in the process by means of an increased coupling with right MCC or with pre-SMA(bilateral); areas whose activity is codifying the uncertainty. Tables with individual seed coordinates based on the Montreal Neurological Institute (MNI) template can be found in Table S5 for MCC(right), Table S6 for pre-SMA(left) and Table S7 for pre-SMA(right).

## Discussion

In this work, Shannon entropy was obtained from the decision outcomes of a group of subjects during an economic decision task under different configurations parametrized by reward probability and time before getting a monetary reward. Among the three different entropy descriptors evaluated, 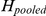 which combines inter and intra-variabilities was the better predictor of the response times. Hence this descriptor was chosen as neural correlate and identified two clusters codifying uncertainty. Although it could be argued that entropies based on either only inter- (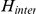) or only within- (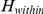) subject variability of decisions would be more intuitive neural correlates, our analyses indicated that response times were slightly better predicted by this pooled approach. Furthermore, this descriptor had a significant contribution to explain response times even for the case of consistent responses, where 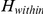 is necessarily zero. This finding indicates that some subjects are consistent in their decisions within task configurations even when the decision becomes difficult and a longer response time is required. In this sense, the desirable tendency to keep self-consistency in responses would be a plausible explanation for this behavior which prevents analysis based on individual outcomes to be the the most appropriate option.

Two clusters whose BOLD activity correlated with the uncertainty associated to each task configuration were found. In particular, a positive linear correlation was found between the activity of these clusters and the Shannon entropy of the pooled decisions reported at each task configuration. A large cluster containing parts of pre-SMA (bilateral) and right MCC and a small cluster located at thalamus (L) were found (see Table 2). These results provide evidence that pre-SMA may cooperate with MCC in codifying and processing uncertainty in decision making. Previous studies have associated medial and or anterior parts of cingulate cortex to decision conflict monitoring and processing. This was obtained by means of activity contrasts between tasks of high and low difficulty guessing [3], high and low conflict measured at the group level [9] or high and low congruency [18] tasks. Our study contributes to better define the modulation of MCC activity in decision making. Rather than obtaining an increased activity in high uncertainty task configurations with respect to low ones, we found that uncertainty is codified within the activity of a cluster that includes right MCC and pre-SMA. In our experiment, the DM
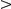
C2 contrast showed a significantly higher activity in this cluster (see Figure S2). This result indicates that the activity codifying uncertainty in both right MCC and pre-SMA(bilateral) is not reflecting motor actions. Furthermore, the connectivity analysis at pre-SMA(bilateral) mainly identified associative areas. Pre-SMA has been implicated in the resolution of conflict, most commonly characterized as an interference between competing motor plans [19], [20]. There is a remarkable difficulty to differentiate motor conflict and decision conflict contributions of this area. While Pochon et al.[9] aimed to uncouple decision conflict from motor conflict and identified a cluster (with similar coordinates to our cluster 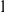 in Table 2), Fortsmann et al. [21] reported that both right pre-SMA and right anterior striatum facilitate fast actions during a decision-making under time pressure. A second cluster was found at the left anterior thalamus. According to the thalamic connectivity atlas [22], is likely to be connected with the pre-frontal cortex (the reported probability was 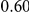).

Two connectivity analyses were carried out to search those areas that gained functional connectivity with right MCC and pre-SMA(bilateral) as the entropy of the task configuration increases. MCC showed functional connectivity with 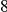 clusters located at the associative cortex within 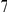 areas (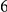 at the frontal lobe and 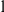 at the temporal lobe). Functional connectivity between the cingulate cortex and frontal and motor areas in a experiment of high versus low congruency has been pointed out [18]. We report here functional connectivity of Pre-SMA(bilateral) with right MCC and with 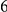 out of its 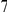 functionally connected areas. In addition, pre-SMA(bilateral) was functionally connected with clusters located in parietal, occipital, and subcortical areas, including well known decision making areas such as MCC and ACC. The insular lobe is also considered to play a key role in emotional decision-making, by means of its reciprocal connectivity with the vmPFC [23], [24], and with the ventral striatum and amygdala [25]. In particular, the posterior insula cortex together with the left caudate nucleus and with the left putamen activity has been associated to choosing delayed relative options instead of immediate rewards [26]. Right middle temporal gyrus is functionally connected with both MCC (cluster 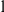) and pre-SMA (cluster 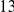). This area has been associated to the action of finding an insight solution to a problem [27], [28]. Left cerebellum VIII was functionally connected to pre-SMA, which has been associated to sensorimotor representation and control [29]. Activity at the dorsolateral and orbital prefrontal cortices have been associated to ventral striatum and thus related to reward and impulsiveness. In particular, they have been implicated in solving decisions under uncertainty [6]–[8]. While activity of these areas was not correlated with Shannon entropy of the decisions, in our experiment they were functionally connected to right MCC and pre-SMA(bilateral). Two possible interpretations can be extracted from a PPI analysis. On the one hand, a psycho-physiological interaction can be seen as a change in the contribution of one area to another due to a change in the psychological variable or context. On the other hand, it can be interpreted as a differential response of an area to the psychological variable which depends on the contribution of a second area [30]. In our case the latter possibility would mean that the more active MCC and pre-SMA are, the more sensitivity of associative areas to depict uncertainty.

The relationship between probability and Shannon entropy is non linear (Figure 1C), being entropy more sensitive as probability reaches its minimum or maximum values. Single-neuron recordings in macaque studies have provided preliminary evidence that such a property may better fit the behavior of dopaminergic neurons under uncertainty paradigms [13], [31]. In our experiment entropy was used as an uncertainty modulator based on the relative frequency of decision outcomes. This approach has permitted to identify the brain areas that codify decision uncertainty and their functional connectivity with other (mainly associative) areas. Therefore Shannon entropy and other information theory measurements should be taken into account as suitable descriptors in cognitive experiments where magnitudes such as conflict, difficulty or uncertainty are aimed to be quantified.

## Materials and Methods

### Subjects

Fifteen undergraduate or graduate students from the University of Navarra were recruited as volunteers for the study. There were seven males and eight females and the mean age was 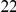 years old (SD 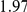). In order to exclude subjects with a current episode or with history of neurological or psychiatric illness, all the volunteers were assessed using The Mini International Neuropsychiatric Interview (MINI) [32] and interviewed about their clinical history by a psychiatrist.

### Ethics statement

The protocol was approved by the ethical committee of the University of Navarra Hospital. Subjects provided written informed consent before entering the scanner.

### Experimental setup

Participants laid supine head first inside the scanner with a four button response box on their abdomen. The middle and index fingers of the right hand and their corresponding buttons were used to choose answers. Experimental stimuli were projected to a mirror over the subject's eyes. Stimulus presentation and response collection were controlled using Cogent 2000 (Wellcome Department of Imaging Neuroscience, UCL, London, UK) and Matlab (The Mathworks, Natick, MA).

### Task

Inside the scanner participants had to repeatedly choose between two options (A and B), which were visually presented. This experiment, including the participants recruitment and the task configurations, was designed according to the probability-time trade-off model within a particular range of paired configurations of probability and time [33]. Each option consisted of an amount of money (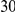 euros) to be received some time in the future with a specific probability. Time (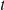) and reward probability (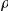) were varied in option B from 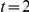 to 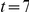 months and from 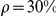 to 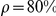, in intervals of 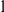 month and 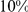 respectively. Option A was maintained in all cases as {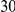 euros,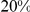, 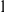 month} (see Figure 1A). Two additional task configurations, with option A not being constant, were formed by {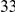 euros,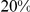,*now*},{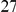 euros,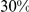,*now*} and {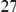 euros,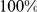,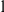 month},{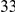 euros,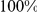,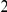 months} respectively. Therefore 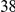 different task configurations were shown to each participant.

Subjects had to choose the option that they considered more attractive using the button box. They were instructed that there were no correct or incorrect answers, and they were not explicitly asked to minimize the time to answer. The time limit to make the decision was fixed to 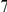 seconds and a white cross was presented one second before the end. There were two control tasks which were interleaved in the presentation (see Figure 1B). They were designed to use almost identical sensory stimulation and required the same motor activity as the decision-making tasks (DM). There were two control tasks. In the attentional control task (C1), subjects had to sum the numbers in each of the options and press the button for the option with the highest result. In the motor control task (C2), options had no numbers but 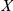 symbols instead (see Figure S4). Subjects were asked to press alternatively one of the buttons every time this control task appeared. C1 was used to check the attentional level of participants and C2 was used to produce a DM
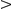
C2 activation map to see areas with activity significantly higher at DM, i.e., activity involved in the decision making task that is not due to motor actions (see Figure S2).

The three conditions (DM,C1 and C2) were grouped in blocks of three trials (see Figure 1B), alternating the experimental task with one of the two control tasks. Three 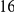-minute scanning sessions were carried out. Sessions consisted of the 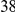 different experimental choices which were repeated 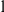 or 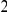 times in a pseudo-random order and the same number of control tasks. Therefore there were twice the number of experimental presentations than of control tasks. Across the 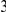 sessions every task configuration was presented 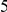 times.

Subjects were awarded with a fixed payment of 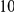 euros. Additionally, they were told that after the experiment one of their choices inside the scanner would be randomly selected and they would have the possibility of receiving the 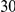 euros with the elected probability and delay. This extra reward was given in order to motivate participants for the task.

### Scanning procedure

The fMRI protocol was carried out with a 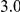 Tesla MR imager (Siemens TRIO, Erlangen, Germany) with a twelve channel head coil. 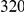 volumes were acquired in every session using T2*-weighted gradient echo-planar imaging (EPI) sequences (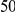 axial slices; slice thickness = 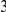 mm; 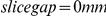; 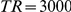 ms; 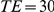 ms; image resolution = 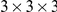 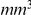; FOV = 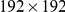 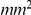; 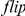 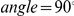). Each time series comprised 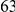 or 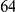 repetitions of the decision-making condition and 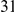 or 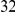 repetitions of each control condition (C1, C2). The anatomical image was 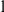 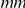 isotropic. A T1-weighted MPRAGE sequence (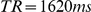, 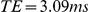, 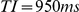, 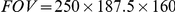 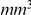, 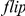 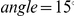, 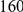 slices) was used for its acquisition.

### Data processing

Data were analyzed using Statistical Parametric Mapping program software (SPM), version 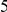 (Wellcome Department of Imaging Neuroscience, UCL, London, UK) in Matlab. For each subject, all EPI volumes were realigned to the first volume of the time series, corrected for differences in the image acquisition time, co-registered with the structural image and spatially normalized into the Montreal Neurological Institute (MNI) template. Finally, a Gaussian smoothing kernel of 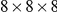 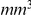 full-width at half maximum was applied to the EPI images.

### Response times

Overall, responses consisted of the 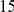 subjects answering 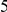 times to each of the 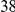 task configurations. Response times (RT) of each of these answers were measured in milliseconds. The average response time per subject 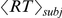 was used as a speed response descriptor of each of the 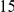 participants. The average response time per subject per task 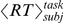 was used to characterize the mean time required by each participant to answer each task configuration and thus contains 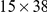 values.

### Shannon entropy

In information theory, Shannon entropy [11] is a measure of the uncertainty associated with a random variable, usually expressed in bits. Its rationale is based on quantifying the amount of information contained in a message. In a more general perspective, the entropy 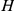 of a discrete random variable 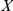 with 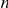 possible values 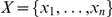 is 
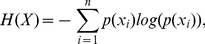
(1)where 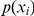 is the probability of 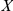 being exactly equal to 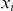 (the probability mass function). In the case of empirical data, 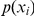 can be estimated by the percent of times that the discrete random variable 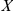 equaled 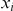. The entropy range of values for any discrete random variable goes from 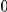 up to 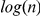 and hence to 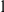 when 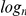 is used. In the case of a binary variable, the maximum entropy is 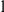 and corresponds, for example, to the entropy of the sequence of outcomes expected when flipping a perfect coin.

Strictly based on the responses obtained, three different entropies 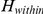, 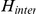 and 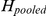 were measured focused on the within-, inter- and pooled-variabilities of the decisions respectively. 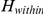 measured, for each task configuration and for each subject, the uncertainty of the 5 decisions given. 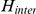 measured, for each task configuration, the uncertainty of the preferred decision of subjects (i.e. the most common response provided among the 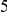 responses given). Finally, 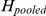 measured, for each task configuration, the uncertainty produced when merging both within- and inter-subject decisions, i.e., when measuring the entropy of the 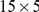 decisions reported for each task configuration.

Every task configuration produces a sequence of binary outcomes that contain the choices made between the constant option A and the alternative option B. Let us define 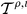 as a task configuration with option A constant and with option B defined by probability 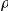 and time 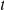. In the particular case of 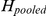, all the decisions made by the participants for each task configuration constitute a dichotomous random variable 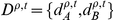. The relative frequencies of choices made by participants at each 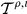 are denoted by 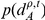 and 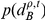 respectively. Hence, according to the equation proposed by C. Shannon, the entropy of each decision variable 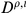 associated to a task configuration 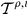 can be defined as 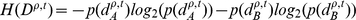. The entropy of a dichotomous random variable is maximal at 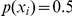 and is highly non linear, being very sensitive to small probability changes near the extremes (when 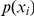 is close to 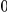 or to 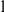). In the case of 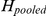, every decision set 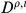 is a random variable consequence of 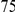 decisions, since every presentation was shown for 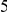 times to each of the 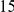 participants. However, in some task configurations where one decision was not made, the entropy was measured according to 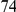 decisions. The entropy model for dichotomous random variables and the values of 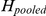 obtained for the task configurations are shown in Figure 1C.

### Multi-linear regression analyses

Three multi-linear regression models were evaluated in order to select which of the three entropies (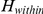, 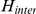 and 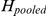) would be used for the neuroimaging analyses. The dependent variable was in all cases 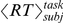. One of the independent variables was 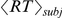, which controlled the possible effects of faster/slower participants. The second independent variable was one of the entropies on each model. For each model, the standardized coefficients of each dependent variable (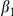, 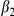) and the 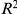 statistic were used to evaluate the goodness of fit were measured. It was hypothesized that, for certain task configurations, a subject could choose every time the same option not only due to a low level of difficulty found but also due to factors such as maintaining self-coherence along the experiment. In this sense, self-coherent answers could still be masking a high level of cognitive conflict. In order to prove such hypothesis one further analysis was carried out analyzing only the 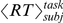 of those answers that conformed consistent decisions per subject per task. Influence of independent variables was considered to be significant with 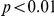.

### Neural correlate analysis of uncertainty

Individual task-related activation was evaluated in a first step using a general linear model. Considering that RT distribution was 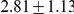, each condition (DM, C1 and C2) was evaluated as event related using a delta function convolved with the hemodynamic response function (canonical HRF). The entropy based modulator (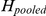) was introduced in the analysis in a later step. This regression model was used to test the areas which showed a positive linear correlation between their BOLD signal and the regressor. Finally, in order to make inferences at the population level, individual contrast images were incorporated into a random effects model [34], [35]. The statistical significance was set at 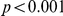 (uncorrected for multiple comparisons). Areas were named according to atlas provided by the SPM anatomy toolbox [36].

### Functional integration analysis of uncertainty

Analysis of functional connectivity assesses the hypothesis that activity in one brain region can be explained by an interaction between the presence of a cognitive process and activity in another part of the brain. In particular, we used the psycho-physiological interactions (PPI) method [30] to estimate functional connectivity with three sources or seeds (MCC(right), pre-SMA(left) and preSMA(right)) during a decision making task whose configurations were labeled by their Shannon entropy (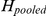). The PPI method is an exploratory multi-regression analysis [37] which includes 4 terms. The psychological variable (Shannon entropy of each task configuration in our case) is the task regressor, the time series of a region (seed) is the physiological variable, a bilinear term formed by the element-by-element product of the task regressor and the seed time series compound the PPI regressor and finally a constant fourth term. The analysis procedure was performed based on [38]. For each subject, three local maxima corresponding to pre-SMA(left and right) and MCC(right) were determined using the individual 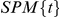 map obtained from the DM
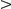
null contrast (coordinates are shown in Tables S6, S7 and S5 respectively). The individual time series for each seed region were obtained by extracting the first principal component from the raw BOLD time series in a spherical ROI (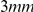 radius) centered on the coordinates of each subject specific local maximum. In a later step, individual level analyses with a separate condition for each task configuration were performed. Within this design, the interaction term (PPI regressor) was estimated. It was computed as the element-by-element product of the time series (for each seed separately) and a Shannon entropy vector coding the uncertainty associated to each task configuration (task regressor). The PPI regressor, the task regressor (psychological variable), the seed time series (physiological variable) and the constant term were introduced as regressors in a first level analysis. At the individual level a 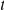-contrast was created using the PPI regressor exclusively. These contrast images were entered into a random effects model [34], [35], followed by a one-sample t-test. The resulting 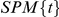 maps were thresholded at 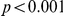 and 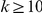. In the case of pre-SMA, a one-way within subject ANOVA with the factor seed, pre-SMA(left) and pre-SMA(right), was performed. Subsequently a conjunction analysis with a conjunction null hypothesis was carried out to find areas common to the two connectivity group maps, i.e., pre-SMA(bilateral).

## Supporting Information

Figure S1
**Individual entropy maps of** 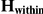 **.** Entropy produced by the 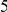 answers reported by each subject to each task configuration. X-axis and Y-axis respectively denote the reward probability and the time to wait of option B. A bilinear interpolation process was applied to the the actual time and probability values evaluated. Color gradient represents the entropy values from 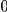 (dark blue) to 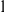 (red). Those maps of subjects with more areas in dark blue correspond to highly self-consistent participants along the whole experiment (e.g. subjects 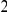, 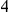 and 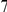).(PDF)

Figure S2

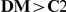
 **contrast task activation.** Blue solid lines indicate MCC(right) with MNI coordinates [12 18 42]. The cluster involving this location contains the largest cluster found to codify decision entropy (cluster 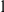 at Table 1 in the manuscript). Therefore neither the activity magnitude nor the activity modulation (correlate with decision entropy) are explained by motor actions.(PDF)

Figure S3
**Areas positively correlated with the entropy values of** 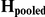 **(** 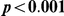 **,** 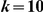 **).** Top. Coronal views. Bottom. Sagital views. The four anatomical regions involved were: middle cingulate cortex (right), pre-supplementary motor area (bilateral), superior medial gyrus (right) and thalamus (left).(PDF)

Figure S4 **Left. Slide corresponding to motor control (C2) events.** Subjects were asked to press alternatively one of the buttons every time this control task appeared. **Right Example of the decision making task (DM).** Subjects were asked to choose the economic option considered more attractive.
(PDF)

Table S1

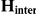
 **: Shannon entropy (bits) for each task configuration** 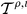 **.** Values are based on the inter-subject variability of the decisions.(PDF)

Table S2

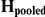
 **: Shannon entropy (bits) for each task configuration** 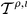 **.** Values are based on the pooled variability (inter- and within-subject) of the decisions.(PDF)

Table S3 **Clusters showing functional connectivity gain with MCC(right) as the entropy increases (PPI analysis).**
(PDF)

Table S4 **Clusters showing functional connectivity gain with pre-SMA(bilateral) as the entropy increases.**
(PDF)

Table S5
**Individual seeds for PPI analysis at the MCC(right).** This table specifies the MNI coordinates used for each subject at MCC(right) and their individual t-values in the DM
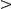
C2 contrast.(PDF)

Table S6
**Individual seeds for PPI analysis at the Pre-SMA (left).** This table specifies the MNI coordinates used for each subject at Pre-SMA(left) and their individual t-values in the DM
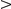
C2 contrast.(PDF)

Table S7
**Individual seeds for PPI analysis at the Pre-SMA (right).** This table specifies the MNI coordinates used for each subject at Pre-SMA(right) and their individual t-values in the DM
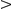
C2 contrast.(PDF)
