# Supplementary figures and images for: The Neural Substrate and Functional Integration of Uncertainty in Decision Making: An Information Theory Approach
Source: PLoS One. 2011 Mar 9;6(3):e17408. doi: 10.1371/journal.pone.0017408 (PMC3052308; doi:10.1371/journal.pone.0017408)

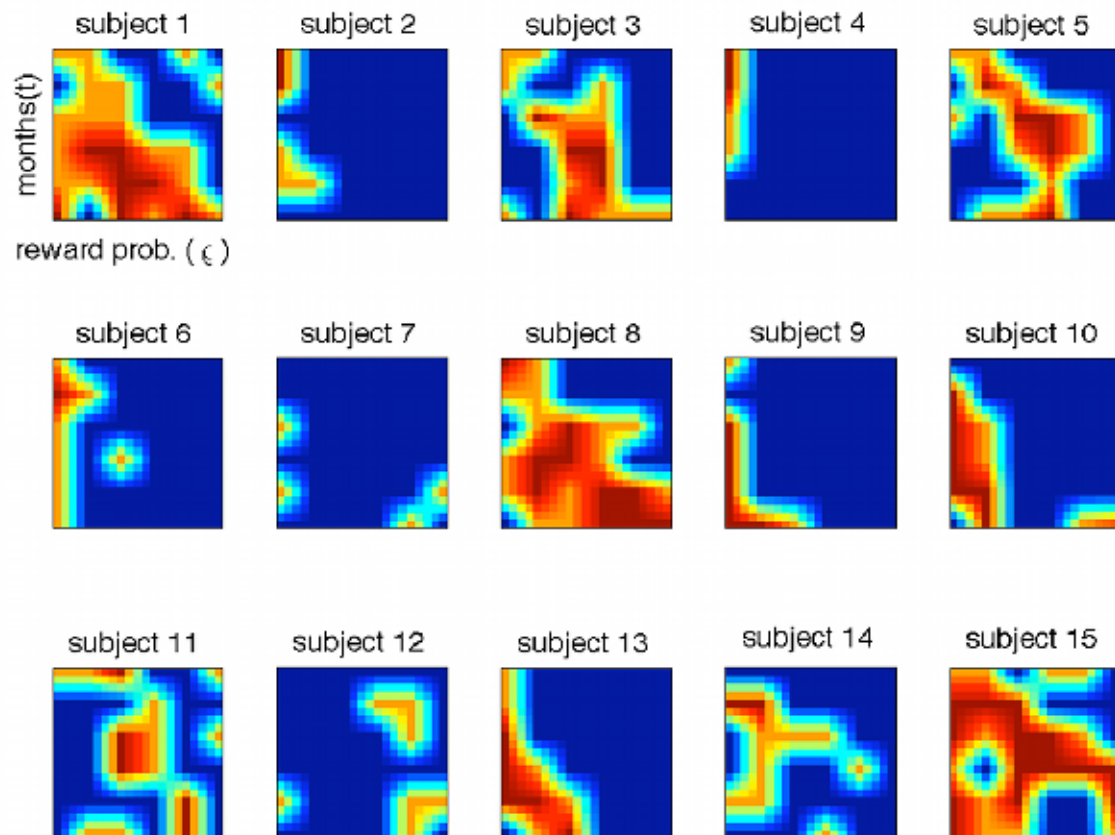

Supplement: Figure S1 — Individual entropy maps of . Entropy produced by the answers reported by each subject to each task configuration. X-axis and Y-axis respectively denote the reward probability and the time to wait of option B. A bilinear interpolation process was applied to the the actual time and probability values evaluated. Color gradient represents the entropy values from (dark blue) to (red). Those maps of subjects with more areas in dark blue correspond to highly self-consistent participants along the whole experiment (e.g. subjects , and ). (PDF) [file pone.0017408.s001.pdf]

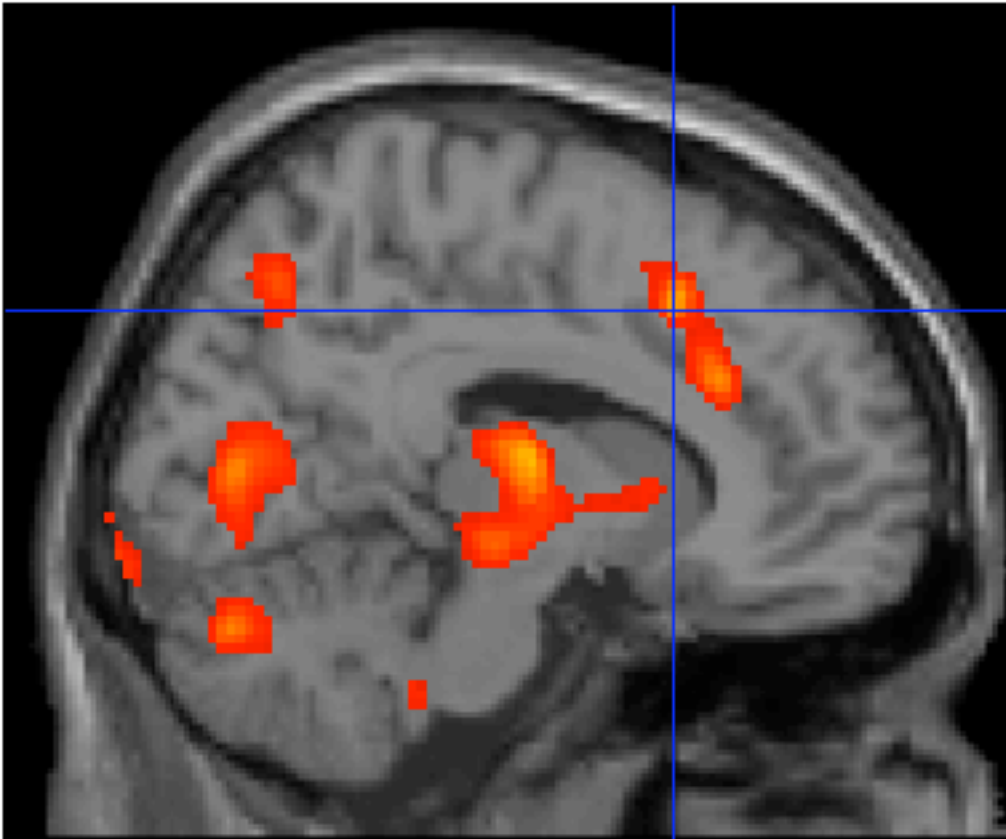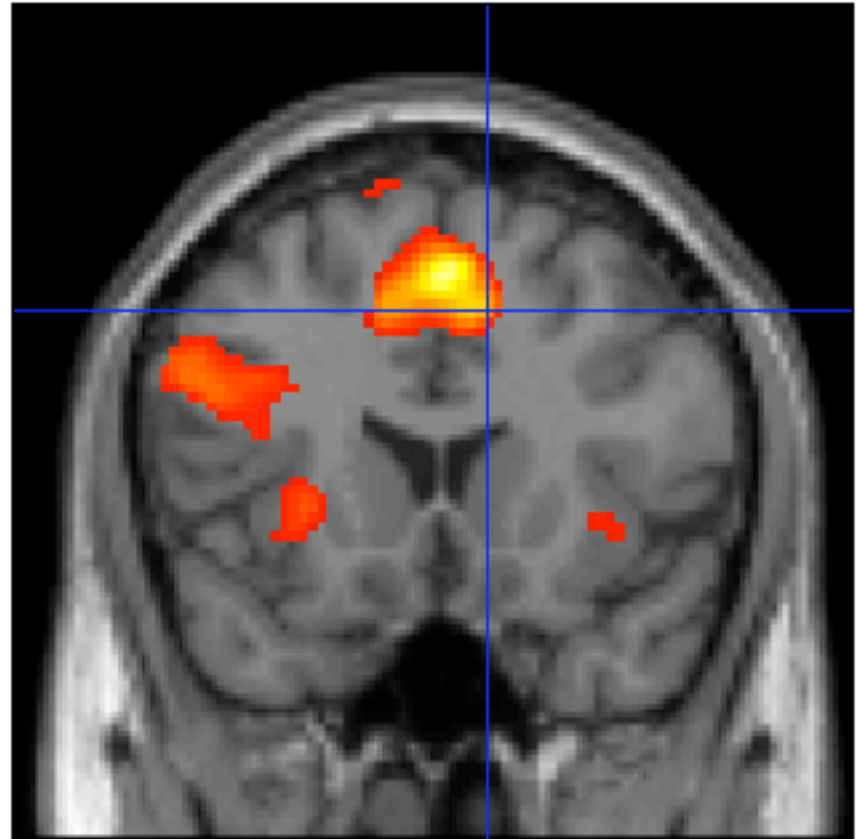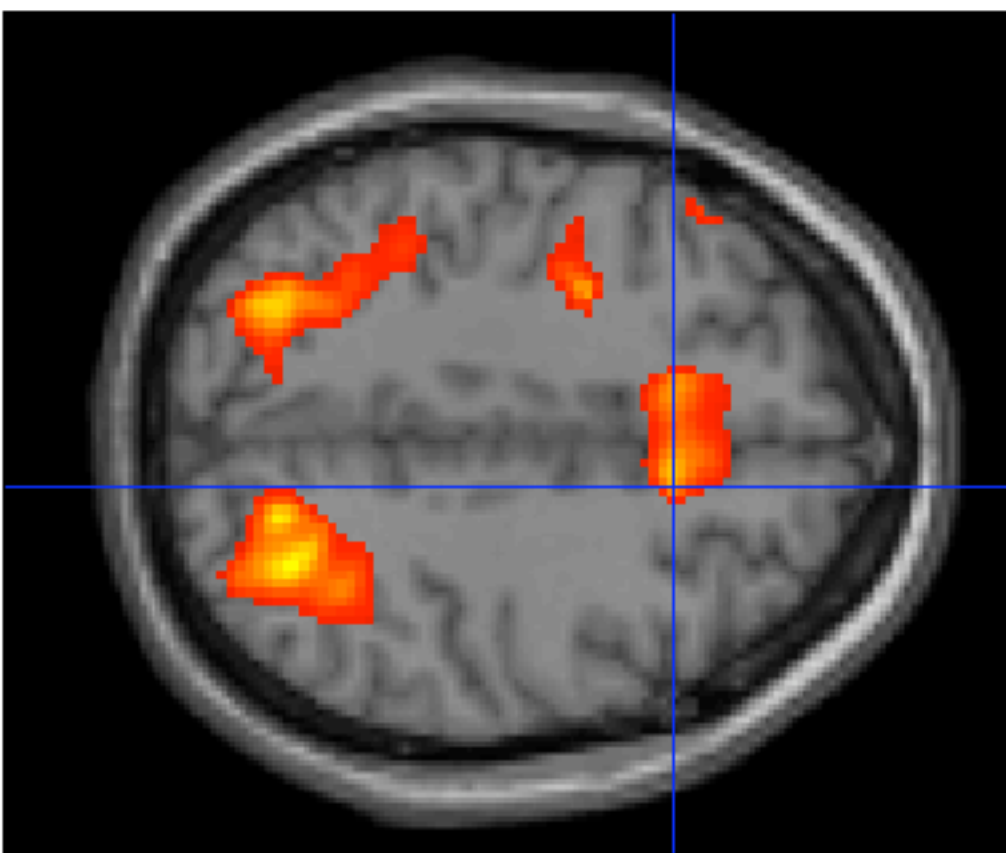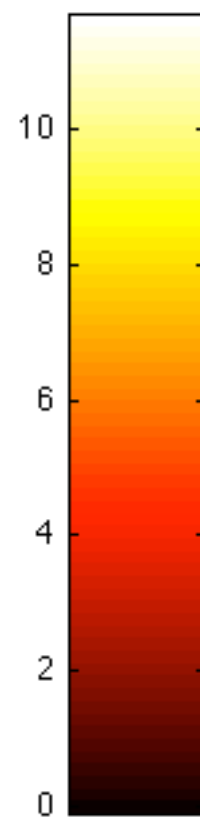

Supplement: Figure S2 — contrast task activation. Blue solid lines indicate MCC(right) with MNI coordinates [12 18 42]. The cluster involving this location contains the largest cluster found to codify decision entropy (cluster at Table 1 in the manuscript). Therefore neither the activity magnitude nor the activity modulation (correlate with decision entropy) are explained by motor actions. (PDF) [file pone.0017408.s002.pdf]

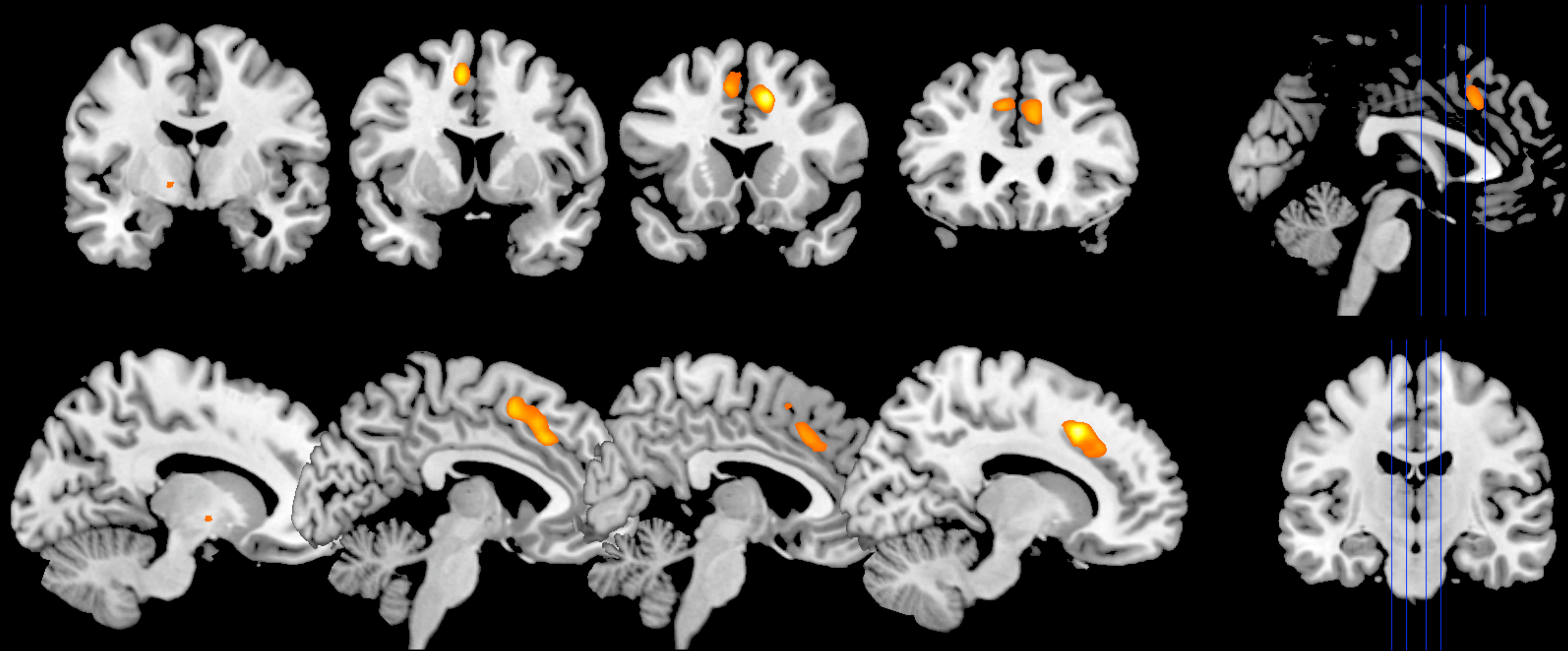

Supplement: Figure S3 — Areas positively correlated with the entropy values of ( , ). Top. Coronal views. Bottom. Sagital views. The four anatomical regions involved were: middle cingulate cortex (right), pre-supplementary motor area (bilateral), superior medial gyrus (right) and thalamus (left). (PDF) [file pone.0017408.s003.pdf]
