# Supplementary material for: The Neural Substrate and Functional Integration of Uncertainty in Decision Making: An Information Theory Approach
Source: PLoS One. 2011 Mar 9;6(3):e17408. doi: 10.1371/journal.pone.0017408 (PMC3052308; doi:10.1371/journal.pone.0017408)
Supplement: Figure S4 — Left. Slide corresponding to motor control (C2) events. Subjects were asked to press alternatively one of the buttons every time this control task appeared. Right Example of the decision making task (DM). Subjects were asked to choose the economic option considered more attractive. (PDF) [file pone.0017408.s004.pdf]

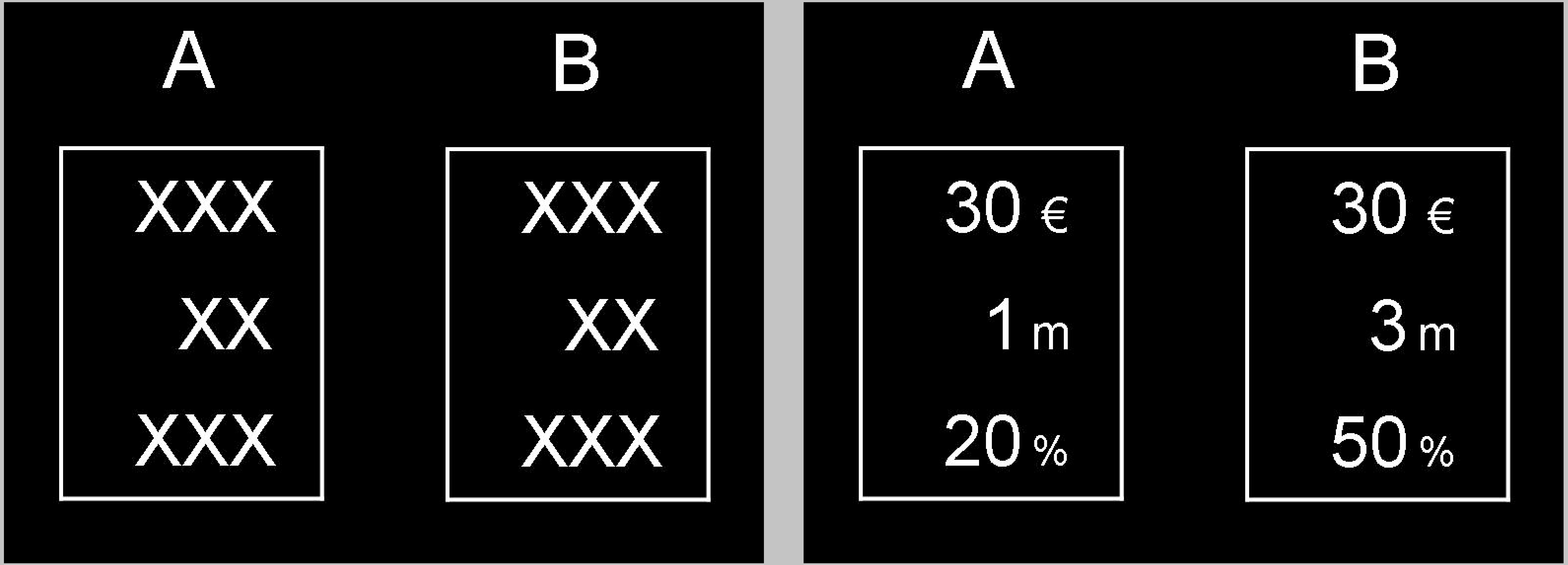
A
B
XXX
XX
XXX
XXX
XX
XXX
A
B
30 €
1 m
20 %
30 €
3 m
50 %
