# Supplementary material for: The Neural Substrate and Functional Integration of Uncertainty in Decision Making: An Information Theory Approach
Source: PLoS One. 2011 Mar 9;6(3):e17408. doi: 10.1371/journal.pone.0017408 (PMC3052308; doi:10.1371/journal.pone.0017408)
Supplement: Table S1 — : Shannon entropy (bits) for each task configuration . Values are based on the inter-subject variability of the decisions. (PDF) [file pone.0017408.s005.pdf]

Table S1. $\mathbf{H_{inter}}$: Shannon entropy (bits) for each task configuration $\mathcal{T}^{\rho,t}$ Values are based on the inter-subject variability of the decisions.

time t	reward probability ρ					
	0.30	0.40	0.50	0.60	0.70	0.80
2	0.9968	0	0	0	0	0
3	0.9710	0.9710	0.3534	0	0	0
4	0.8366	0.9968	0.9183	0.5665	0.3534	0
5	0.5665	0.9968	0.9710	0.8366	0.7219	0.5665
6	0.5665	0.9183	0.9710	0.9710	0.8366	0.7219
7	0.3534	0.9183	0.9968	0.9183	0.9183	0.7219

Entropy for {27 euros,100%,1 month} and {33 euros,100%,2 months} is 0.7219 bits.
Entropy for {33 euros,20%,*now*} and {27 euros,30%,*now*} is 0.9183 bits.
