# Supplementary material for: The Neural Substrate and Functional Integration of Uncertainty in Decision Making: An Information Theory Approach
Source: PLoS One. 2011 Mar 9;6(3):e17408. doi: 10.1371/journal.pone.0017408 (PMC3052308; doi:10.1371/journal.pone.0017408)
Supplement: Table S2 — : Shannon entropy (bits) for each task configuration . Values are based on the pooled variability (inter- and within-subject) of the decisions. (PDF) [file pone.0017408.s006.pdf]

Table S2. $\mathbf{H_{pooled}}$: Shannon entropy (bits) for each task configuration $\mathcal{T}^{\rho,t}$. Values are based on the pooled variability (inter- and within-subject) of the decisions.

time t	reward probability ρ					
	0.30	0.40	0.50	0.60	0.70	0.80
2	0.9953	0.4022	0.1774	0.1033	0.1774	0.1022
3	0.9427	0.9626	0.5294	0.2423	0.1022	0.1022
4	0.8893^A	0.9968	0.8730	0.6944	0.4475	0.1022
5	0.7478^A	0.9999	0.9532	0.9183	0.6944	0.6343
6	0.7722^A	0.9896^A	0.9809	0.9782	0.8366	0.6705
7	0.5294^A	0.9710^A	0.9937	0.9311	0.9183	0.8218

Entropy for {27 euros,100%,1 month} and {33 euros,100%,2 months} is 0.8780 bits.
Entropy for {33 euros,20%,*now*} and {27 euros,30%,*now*} is 0.9626 bits.
Exponent A denotes that option A was preferred to option B (no exponent otherwise).
