# Supplementary material for: The Neural Substrate and Functional Integration of Uncertainty in Decision Making: An Information Theory Approach
Source: PLoS One. 2011 Mar 9;6(3):e17408. doi: 10.1371/journal.pone.0017408 (PMC3052308; doi:10.1371/journal.pone.0017408)
Supplement: Table S3 — Clusters showing functional connectivity gain with MCC(right) as the entropy increases (PPI analysis). (PDF) [file pone.0017408.s007.pdf]

Table S3. Clusters showing functional connectivity gain with MCC(right) as the entropy increases (PPI analysis).

Cluster	voxels	Anatomical area (side)	Lobe	MNI coordinates			t-value
				x	y	z	
1	147	Middle Temporal Gyrus (R)	Temporal	62	-6	-24	5.91
2	120	Middle Frontal Gyrus (L)	Frontal	-24	24	46	5.35
		Superior Frontal Gyrus (L)	Frontal	-16	30	40	4.68
3	44	Middle Temporal Gyrus (L)	Temporal	-64	-26	-4	5.27
4	27	Superior Medial Gyrus (L)	Frontal	-4	64	12	4.37
		Superior Frontal Gyrus (L)	Frontal	-12	66	14	4.20
5	25	Superior Medial Gyrus (R)	Frontal	10	62	2	5.20
6	17	Middle Orbital Gyrus (L)	Frontal	-8	54	-2	5.06

Height threshold t-value=3.85, $p < 0.001$ uncorrected. Extent threshold: $k = 10$ voxels.
MNI coordinates of seed at MCC(R): [12 18 42].
