# Supplementary material for: The Neural Substrate and Functional Integration of Uncertainty in Decision Making: An Information Theory Approach
Source: PLoS One. 2011 Mar 9;6(3):e17408. doi: 10.1371/journal.pone.0017408 (PMC3052308; doi:10.1371/journal.pone.0017408)
Supplement: Table S4 — Clusters showing functional connectivity gain with pre-SMA(bilateral) as the entropy increases. (PDF) [file pone.0017408.s008.pdf]

Table S4. Clusters showing functional connectivity with pre-SMA(bilateral) as the entropy increases.

Cluster	voxels	Anatomical area (side)	Lobe	MNI coordinates			t-value
				x	y	z	
1	902	Precuneus (R)	Parietal	12	-46	62	4.51
		Superior Parietal Lobule (R)	Parietal	26	-50	68	4.25
		Postcentral Gyrus (R)	Parietal	26	-30	60	4.06
		Middle Cingulate Cortex (L)	Frontal	-2	-28	46	3.81
		Paracentral Lobule (R)	Frontal	14	-32	50	3.64
		Middle Cingulate Cortex (R)	Frontal	14	-30	46	3.53
2	130	Posterior Insula (R)	Insular	40	-6	-8	4.34
3	120	Superior Parietal Lobule (L)	Parietal	-26	-46	66	3.84
		Postcentral Gyrus (L)	Parietal	-28	-38	50	3.77
4	96	Temporal Pole (R)	Temporal	62	4	-2	4.18
5	62	Middle Frontal Gyrus (L)	Frontal	-20	22	44	3.62
6	60	Cerebelum VIII (L)	Cerebelum	-20	-46	-56	5.03
7	55	Middle Cingulate Cortex (R)	Frontal	8	-8	46	3.72
8	42	Anterior Cingulate Cortex (L)	Frontal	-4	50	0	3.83
9	38	Superior Medial Gyrus (R)	Frontal	10	60	4	4.12
10	34	Precentral Gyrus (R)	Frontal	46	-14	62	4.11
11	32	Middle Temporal Gyrus (L)	Temporal	-54	-68	18	4.16
12	24	Caudate Nucleus (L)	Basal Ganglia	-6	14	-4	4.31
13	23	Middle Temporal Gyrus (R)	Temporal	60	-12	-20	3.94
14	21	Superior Temporal Gyrus (R)	Temporal	58	-60	22	3.80
15	20	Paracentral Lobule (L)	Frontal	-16	-40	54	3.68
16	17	Postcentral Gyrus (L)	Parietal	-26	-32	72	3.82
17	14	Superior Medial Gyrus (L)	Frontal	-2	64	10	3.60
18	13	Superior Frontal Gyrus (L)	Frontal	-18	14	48	3.62
19	11	Pulvinar (L)	Thalamus	-14	-34	2	3.80

This a analysis is the result of a conjunction of PPIs seeded in pre-SMA(L) and pre-SMA(R).
Height threshold: t-value=3.31, $p < 0.001$ uncorrected. Extent threshold: $k = 10$ voxels.
MNI coordinates of seed at pre-SMA(L): [-8 8 52]. MNI coordinates of seed at pre-SMA(R): [6 12 54].
