# Supplementary material for: The Neural Substrate and Functional Integration of Uncertainty in Decision Making: An Information Theory Approach
Source: PLoS One. 2011 Mar 9;6(3):e17408. doi: 10.1371/journal.pone.0017408 (PMC3052308; doi:10.1371/journal.pone.0017408)
Supplement: Table S5 — Individual seeds for PPI analysis at the MCC(right). This table specifies the MNI coordinates used for each subject at MCC(right) and their individual t-values in the DMC2 contrast. (PDF) [file pone.0017408.s009.pdf]

Table S5. Individual seeds for PPI analysis at the MCC(right). This table specifies the MNI coordinates used for each subject at MCC(right) and their individual t-values in the DM>C2 contrast.

subject	local maxima			DM>C2
id	x	y	z	t-value
1	8	24	44	5.16
2	6	14	50	4.45
3	8	24	42	9.88
4	6	24	44	10.80
5	4	14	48	7.45
6	4	12	50	7.48
7	8	10	50	9.89
8	-	-	-	-
9	8	14	48	5.95
10	8	22	42	10.93
11	8	12	50	5.82
12	8	14	50	10.38
13	2	22	42	9.80
14	6	14	44	9.48
15	8	20	38	7.93

DM>C2 local maxima coordinates and t-values for the contrast of each subject. Subject number 8 did not show DM>C2 activations around these coordinates and therefore was not included in this analysis. Coordinates at the group level are [12 18 42] (t-value=7.72)
