# Supplementary material for: The Neural Substrate and Functional Integration of Uncertainty in Decision Making: An Information Theory Approach
Source: PLoS One. 2011 Mar 9;6(3):e17408. doi: 10.1371/journal.pone.0017408 (PMC3052308; doi:10.1371/journal.pone.0017408)
Supplement: Table S6 — Individual seeds for PPI analysis at the Pre-SMA (left). This table specifies the MNI coordinates used for each subject at Pre-SMA(left) and their individual t-values in the DMC2 contrast. (PDF) [file pone.0017408.s010.pdf]

Table S6. Individual seeds for PPI analysis at the Pre-SMA (left). This table specifies the MNI coordinates used for each subject at Pre-SMA(left) and their individual t-values in the DM>C2 contrast.

subject	local maxima			DM>C2
id	x	y	z	t-value
1	-6	14	48	6.21
2	-10	10	58	6.65
3	-6	20	54	10.23
4	-4	12	52	9.44
5	-6	8	56	8.29
6	-6	14	52	7.21
7	-4	6	50	7.68
8	-8	12	50	5.52
9	-10	10	44	6.42
10	-8	8	52	13.68
11	-6	10	50	4.94
12	-4	10	50	12.42
13	-6	8	54	8.27
14	-4	12	48	9.35
15	-6	8	54	12.85

DM>C2 local maxima coordinates and t-values for contrast of each subject.
Coordinates at the group level are [-8 8 52] (t-value=6.07).
