# Supplementary material for: The Neural Substrate and Functional Integration of Uncertainty in Decision Making: An Information Theory Approach
Source: PLoS One. 2011 Mar 9;6(3):e17408. doi: 10.1371/journal.pone.0017408 (PMC3052308; doi:10.1371/journal.pone.0017408)
Supplement: Table S7 — Individual seeds for PPI analysis at the Pre-SMA (right). This table specifies the MNI coordinates used for each subject at Pre-SMA(right) and their individual t-values in the DMC2 contrast. (PDF) [file pone.0017408.s011.pdf]

Table S7. Individual seeds for PPI analysis at the Pre-SMA (right). This table specifies the MNI coordinates used for each subject at Pre-SMA(right) and their individual t-values in the DM>C2 contrast.

subject	local maxima			DM>C2
id	x	y	z	t-value
1	4	16	48	5.42
2	6	14	50	4.45
3	6	14	56	4.86
4	6	14	50	5.49
5	6	14	54	8.43
6	4	12	50	7.48
7	8	10	50	9.89
8	4	12	54	4.68
9	8	14	48	5.95
10	6	12	54	11.41
11	8	12	50	5.82
12	8	14	50	10.38
13	4	10	54	5.94
14	6	14	50	9.28
15	6	10	54	12.34

DM>C2 local maxima coordinates and t-values for each subject.
Coordinates at the group level are [6 12 54] (t-value=4.04).
